# Outcome prediction of head and neck squamous cell carcinoma by MRI radiomic signatures

**DOI:** 10.1007/s00330-020-06962-y

**Published:** 2020-06-04

**Authors:** Steven W. Mes, Floris H. P. van Velden, Boris Peltenburg, Carel F. W. Peeters, Dennis E. te Beest, Mark A. van de Wiel, Joost Mekke, Doriene C. Mulder, Roland M. Martens, Jonas A. Castelijns, Frank A. Pameijer, Remco de Bree, Ronald Boellaard, C. René Leemans, Ruud H. Brakenhoff, Pim de Graaf

**Affiliations:** 1 Otolaryngology – Head and Neck Surgery, Cancer Center Amsterdam, Amsterdam UMC, Vrije Universiteit Amsterdam, Amsterdam, The Netherlands; 2 Department of Radiology, Section of Nuclear Medicine, Leiden University Medical Center, Leiden, The Netherlands; 3 Department of Head and Neck Surgical Oncology, University Medical Center Utrecht, Utrecht, The Netherlands; 4 Epidemiology & Biostatistics, Amsterdam Public Health Research Institute, Amsterdam UMC, Vrije Universiteit Amsterdam, Amsterdam, The Netherlands; 5 Biometris, Wageningen University & Research, Wageningen, Netherlands; 6 MRC Biostatistics Unit, Cambridge University, Cambridge, UK; 7 Department of Oral and Maxillofacial Surgery, Northwest Clinics Alkmaar, Alkmaar, The Netherlands; 8 Radiology and Nuclear Medicine, Cancer Center Amsterdam, Amsterdam UMC, Vrije Universiteit Amsterdam, De Boelelaan 1117, 1081 HV Amsterdam, The Netherlands; 9 Department of Radiology, University Medical Center Utrecht, Utrecht, The Netherlands

**Keywords:** Magnetic resonance imaging, Head and neck neoplasms, Prognosis, Factor analysis

## Abstract

**Objectives:**

Head and neck squamous cell carcinoma (HNSCC) shows a remarkable heterogeneity between tumors, which may be captured by a variety of quantitative features extracted from diagnostic images, termed radiomics. The aim of this study was to develop and validate MRI-based radiomic prognostic models in oral and oropharyngeal cancer.

**Materials and Methods:**

Native T1-weighted images of four independent, retrospective (2005–2013), patient cohorts (*n* = 102, *n* = 76, *n* = 89, and *n* = 56) were used to delineate primary tumors, and to extract 545 quantitative features from. Subsequently, redundancy filtering and factor analysis were performed to handle collinearity in the data. Next, radiomic prognostic models were trained and validated to predict overall survival (OS) and relapse-free survival (RFS). Radiomic features were compared to and combined with prognostic models based on standard clinical parameters. Performance was assessed by integrated area under the curve (iAUC).

**Results:**

In oral cancer, the radiomic model showed an iAUC of 0.69 (OS) and 0.70 (RFS) in the validation cohort, whereas the iAUC in the oropharyngeal cancer validation cohort was 0.71 (OS) and 0.74 (RFS). By integration of radiomic and clinical variables, the most accurate models were defined (iAUC oral cavity, 0.72 (OS) and 0.74 (RFS); iAUC oropharynx, 0.81 (OS) and 0.78 (RFS)), and these combined models outperformed prognostic models based on standard clinical variables only (*p* < 0.001).

**Conclusions:**

MRI radiomics is feasible in HNSCC despite the known variability in MRI vendors and acquisition protocols, and radiomic features added information to prognostic models based on clinical parameters.

**Key Points:**

*• MRI radiomics can predict overall survival and relapse-free survival in oral and HPV-negative oropharyngeal cancer.*

*• MRI radiomics provides additional prognostic information to known clinical variables, with the best performance of the combined models.*

*• Variation in MRI vendors and acquisition protocols did not influence performance of radiomic prognostic models.*

**Electronic supplementary material:**

The online version of this article (10.1007/s00330-020-06962-y) contains supplementary material, which is available to authorized users.

## Introduction

Head and neck squamous cell carcinoma (HNSCC) is a malignancy arising in the mucosal lining of the oral cavity, oropharynx, larynx, and hypopharynx [1]. Unfortunately, mortality rates are high [2], and long-term functional deficits often remain after therapy [3]. Ideally, treatment is personalized to maximize treatment efficacy and minimize side effects. However, treatment personalization is currently only based on stage, site, and histological parameters after surgery, with suboptimal performance [4].

Despite that HNSCC arise in one tissue type, they are remarkably heterogeneous hampering accurate prediction of clinical behavior [5]. This heterogeneous tumor biology may be captured by imaging [6, 7]. In the past, images were mostly described by qualitative features such as dimension and invasion in neighboring structures, but currently images are also being analyzed by extraction of a variety of quantitative features, also termed radiomics [8].

Radiomic analyses have previously been applied in HNSCC patients, but most studies focused on computed tomography (CT), most particularly for radiotherapy planning. Aerts et al described a prognostic radiomic signature based on CT scans of lung cancer and applied this signature successfully in oropharyngeal cancer [9]. Others followed with comparable approaches [10–14]. The preference for CT is explained by (i) intuitive interpretation of signal intensities that correspond to tissue radiodensity [8], (ii) standardization of imaging performance across vendors and scanners [8], and (iii) availability of delineated tumor volumes from radiation treatment plans.

Nonetheless, in clinical practice, magnetic resonance imaging (MRI) is often the modality of choice for imaging of head and neck tumors, because of the superior soft tissue contrast. However, the acquired MRI signal intensities are influenced by scanner parameters and many image acquisition-related factors [15]. Still, MRI can identify physical properties of the tumor by application of separate sequence acquisition protocols (e.g., diffusion-weighted MRI (DWI), dynamic contrast-enhanced (DCE) MRI [16]), and therefore, MRI might better capture overall tumor biology than CT. As such, MRI radiomics was able to categorize breast cancer, glioblastoma, and prostate cancer in different molecular subtypes [17–19]. In HNSCC, prognostic models based on MRI radiomics were only described for small series of less than 20 cases of oropharyngeal cancer [20, 21] or heterogeneous cohorts [22, 23].

In this study, we present an MRI radiomics workflow based on T1-weighted images that is applied in two independent patient cohorts of oral cancer (*n* = 102 and *n* = 76) and two cohorts of HPV-negative oropharyngeal cancer (*n* = 89 and *n* = 56) for prediction of overall survival (OS) and relapse-free survival (RFS).

## Material and methods

### Patients

Four independent, retrospective cohorts of HNSCC patients included (i) a cohort of oral squamous cell carcinoma (OSCC) patients from Amsterdam UMC, location VUmc (VUMC), treated from 2005 to 2013; (ii) a cohort of OSCC patients from University Medical Center Utrecht (UMCU) treated from 2010 to 2013; (iii) a cohort of HPV-negative oropharyngeal squamous cell carcinoma (OPSCC) patients from VUMC, treated from 2008 to 2012; and (iv) a cohort of HPV-negative OPSCC patients from UMCU treated from 2010 to 2013. All patients were treated with curative intent. HPV status was assessed with p16 immunohistochemistry and subsequent PCR-based HPV DNA detection on p16-immunopositive cases. HPV-positive tumors were excluded because this group is considered to be a separate disease entity within HNSCC [24], which would interfere with radiomic findings [25] and clinical outcome [26]. The Dutch Medical Research Involving Human Subjects Act (WMO) does not apply to this study and therefore informed consent was waived by the Medical Ethics Review Committee at Amsterdam UMC. Medical records were reviewed to obtain clinical characteristics, including age at diagnosis, gender, comorbidity, and clinical TNM-stage (7th edition) [27]. Comorbidity was classified using the Adult Comorbidity Evaluation 27 (ACE-27) [28]. Two outcome measures were used: (a) OS, which was defined as time from date of incidence to death from any cause; and (b) RFS, which was defined as time from date of incidence to development of locoregional recurrence, distant metastasis, or second primary HNSCC. For RFS, patients who died of other causes or developed other tumors outside the head and neck region were censored at the date of death or incidence date of the other tumor.

### MRI

**Fig. 1:**
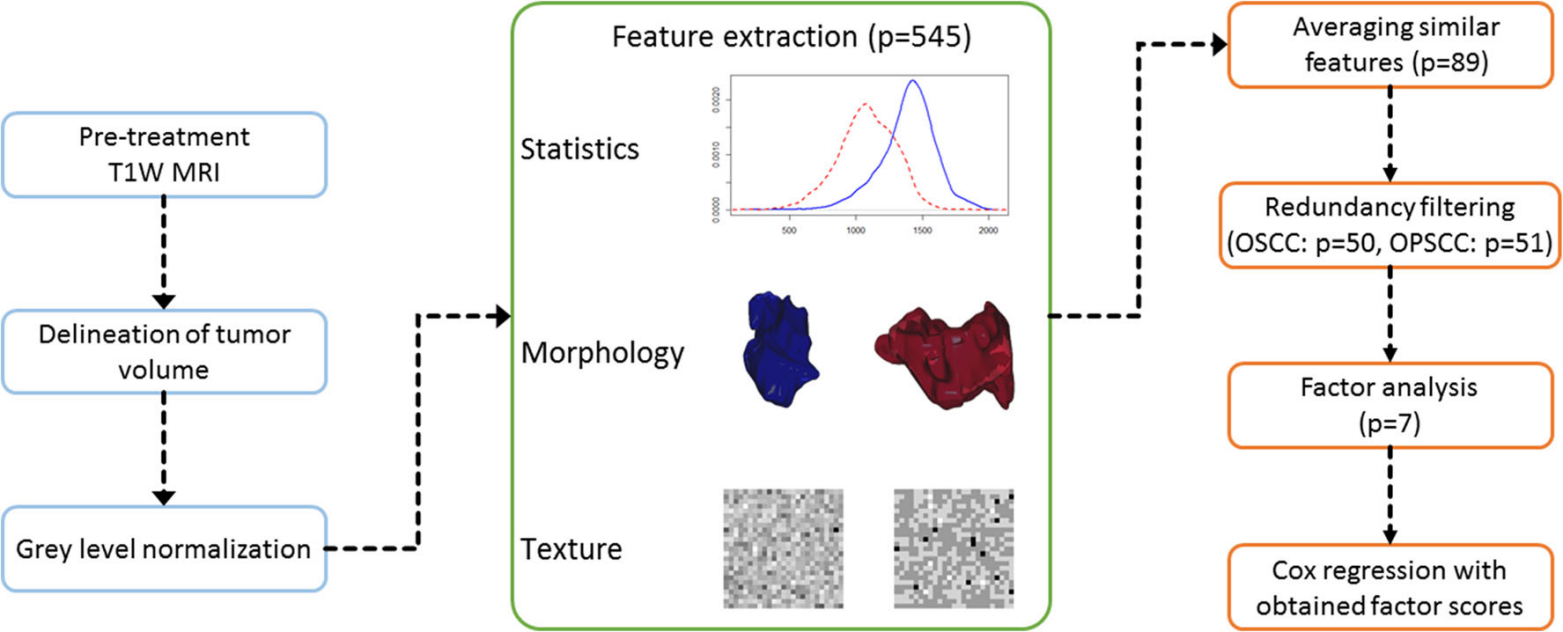
Illustration of radiomics pipeline. Abbreviations: MRI, magnetic resonance imaging; OPSCC, oropharyngeal squamous cell carcinoma; OSCC, oral cavity squamous cell carcinoma; T1W, T1-weighted

The schematic workflow of this study is depicted in Fig. 1. Axial 2D T1W images without gadolinium enhancement and short TI inversion recovery (STIR) (OSCC VUMC, OSCC UMCU, OPSCC VUMC) or T2-weighted (OPSCC UMCU) images were available for all patients. These scans were obtained using scanners of different vendors and protocols (Supplemental Table 1). Native T1W images were used for feature extraction because this sequence was available for all tumors. The STIR sequence was used to facilitate tumor segmentation, and for feature extraction in the OSCC cohorts to assess a possible additional prognostic value. Our protocols of contrast-enhanced T1W imaging changed in time (e.g., slice thickness, 2D versus 3D, with or without fat saturation), and therefore this sequence was not considered in this study.

### Segmentation

**Fig. 2:**
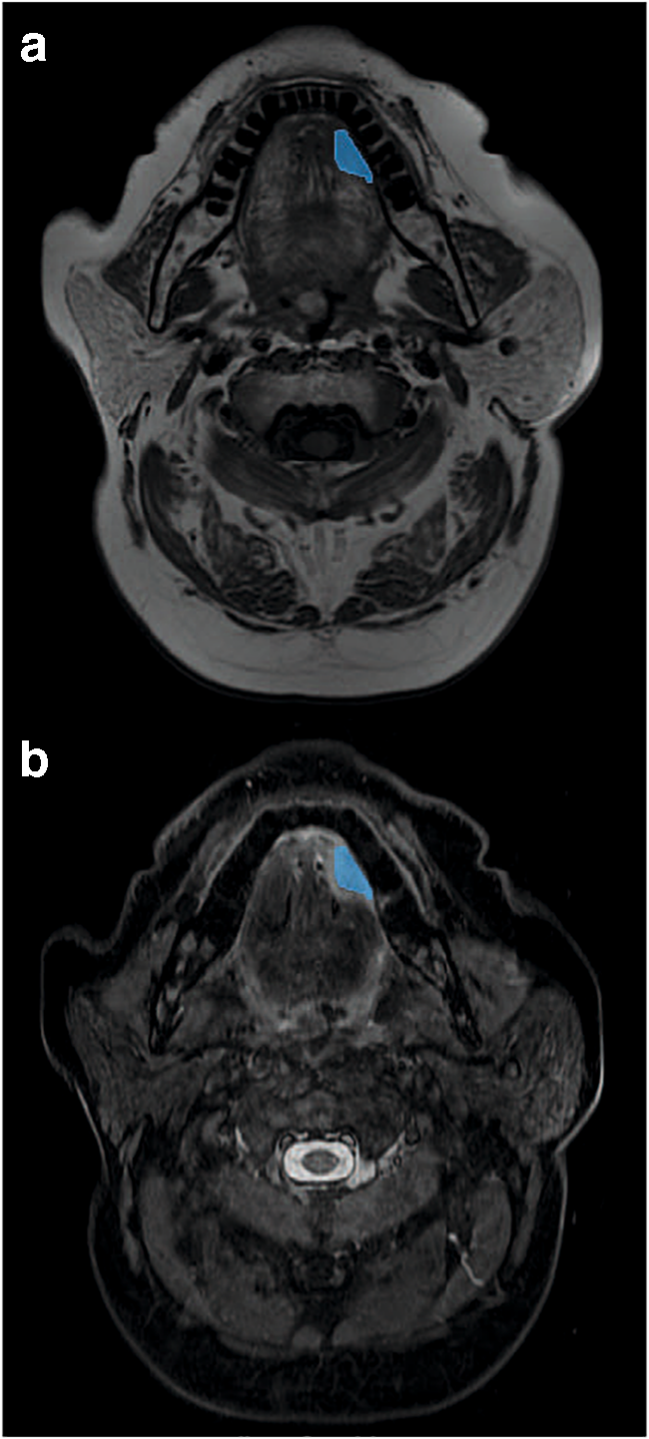
Illustration of tumor segmentation on T1 MRI and STIR. Exemplary segmentation of a T2N2b tongue tumor on the left side on T1W MRI (**a**) and STIR (**b**)

MR images of VUMC patients were transferred to VelocityAI 3.1 (Varian Medical Systems), whereas UMCU MRI scans were transferred to an in-house developed target volume delineation tool [29]. Subsequently, STIR images were automatically co-registered to the T1W images and registration was visually checked. Supervised manual delineation of all primary tumors was performed by S.M. and B.P. (both 3 years of experience) with visual inspection of delineation by senior head and neck radiologists (P.G. or F.P. with 11 and 25 years of experience). In Fig. 2, an example of a delineated tumor is shown on T1W MRI and STIR.

### Feature extraction and processing

The feature extraction and processing can be found in detail in the Supplemental Methods. The extracted features are described in Table 1.

**Table 1:** Radiomic raw features (*p* = 545)

Group	Number	Name
First-order statistics	35	From entire image (before normalization): maximum gray level, minimum gray level, range, mean, median, standard deviation, maximum gray level of all values over 0.5, median of all values over 0.5, mean of all values over 0.5 From tumor VOI (after normalization): maximum gray level, minimum gray level, range, mean, median, standard deviation, interquartile range, coefficient of variation (COV, in percentage), skewness, kurtosis, excess kurtosis, median absolute deviation of the median, mean absolute deviation of the median, mean absolute deviation of the mean, mean Laplacian, total energy, variance, root-mean-square (RMS), mean of the maximum voxel and the six adjacent voxels (Max_star_), integrated intensity, entropy^a^, uniformity^a^
Spatial autocorrelation	2	Moran’s I, Geary’s C
Intensity-volume histogram features	1	Area under a cumulative intensity-volume histogram curve (AUC)
Morphological features	11	Tumor volume, surface area, surface-to-volume ratio, surface area to surface of an equivolumetric sphere-to-volume ratio, radius of an equivolumetric sphere, compactness 1, compactness 2, spherical disproportion, sphericity, asphericity, maximum 3D diameter
Fractal features	4	Fractal dimension (calculated), fractal dimension (fitted), fractal abundance, fractal lacunarity
Texture features based on gray level co-occurrence matrix^a,b^	300	Joint maximum, joint average, joint variance, joint entropy, difference average, difference variance, difference entropy, sum average, sum variance, sum entropy, angular second moment, contrast, dissimilarity, inverse difference, inverse difference normalized, inverse difference moment, inverse difference moment normalized, inverse variance, correlation, autocorrelation, cluster tendency, cluster shade, cluster prominence, first measure of information correlation, second measure of information correlation
Texture features based on gray level run length^a,b^	192	Short-run emphasis, long-run emphasis, low-gray-level-run emphasis, high-gray-level-run emphasis, short-run low-gray-level emphasis, short-run high-gray-level emphasis, long-run low-gray-level emphasis, long-run high-gray-level emphasis, gray level non-uniformity, gray level non-uniformity normalized, run length non-uniformity, run length non-uniformity normalized, run percentage, gray level variance, run length variance, run entropy

^a^Obtained using a discretization of 32, 64, or 128 gray level bins

^b^Calculated from matrices per direction and then averaged (average), or from merged matrix created using all matrices over all directions (combined). The matrices were calculated either per *x*-*y* plane (2D, but all planes were used in the calculation) or volumetrically (3D)

### Interobserver feature stability

MRI scans of 30 OPSCCs were re-segmented by an independent senior head and neck radiologist (J.C., with 35 years of experience) according to the pipeline described before. Subsequently, feature extraction was performed and the mean value of similar features was determined, leaving *n* = 89 unique features. The Kendall’s coefficient of concordance was determined and a coefficient of ≥ 0.7 was considered high concordance.

### Factor analysis and model training

The subsequent steps of predictive modelling that were applied in this study have been described before [30], and can be found in detail in the Supplemental Methods.

### Influence of vendor and magnetic field strength

As described above, a variety of MRI acquisition protocols and equipment of different vendors were used. Although this may impact the radiomics analyses, it reflects current clinical routine. Ideally, a correlation analyses would be performed of test-retest data from different vendors and magnetic field strengths to standardize the data, but such datasets are not available. Instead, multivariate analysis of variance (MANOVA) was performed to compare the mean factor scores between vendors and magnetic field strength in VUMC patient cohorts. In the UMCU cohorts (OSCC and OPSCC), only the mean factor scores between magnetic field strengths were compared, because most scans were obtained using one MR vendor (Table 2).

**Table 2:** Patient characteristics

		VUMC OSCC	UMCU OSCC	VUMC OPSCC	UMCU OPSCC	*p* value*	*p* value±
Number of cases		102	76	89	56		
Median age	Years (MAD)	63 (11.9)	66.3 (11.1)	60 (7.4)	64 (11.9)	0.23	0.24
Gender	Male	64 (62.7)	46 (60.5)	49 (55.1)	35 (62.5)		
	Female	38 (37.3)	30 (39.5)	40 (44.9)	21 (37.5)	0.77	0.48
Smoking	Current	51 (50.0)	34 (44.7)	54 (60.7)	34 (60.7)		
	Former	35 (34.3)	24 (31.6)	26 (29.2)	13 (23.2)		
	Never	16 (15.7)	15 (19.7)	9 (10.1)	6 (10.7)		
	Unknown	0 (0)	3 (3.9)	0 (0)	3 (5.4)	0.23	0.16
Alcohol	Current	68 (66.7)	49 (64.5)	66 (74.2)	40 (71.4)		
	Former	13 (12.7)	6 (7.9)	12 (13.5)	10 (17.9)		
	Never	21 (20.6)	17 (22.4)	11 (12.4)	3 (5.4)		
	Unknown	0 (0)	4 (5.3)	0 (0)	3 (5.4)	0.11	0.07
ACE27	0	28 (27.5)	27 (35.5)	26 (29.2)	17 (30.4)		
	1	34 (33.3)	40 (52.6)	33 (37.1)	27 (48.2)		
	2	28 (27.5)	4 (5.3)	27 (30.3)	7 (12.5)		
	3	12 (11.8)	5 (6.6)	3 (3.4)	1 (1.8)		
	Unknown	0 (0)	0 (0)	0 (0)	4 (7.1)	< 0.001	0.01
T-stage	1	12 (11.8)	20 (26.3)	7 (7.9)	6 (10.7)		
	2	36 (35.3)	28 (36.8)	35 (39.3)	17 (30.4)		
	3	21 (20.6)	4 (5.3)	16 (18.0)	13 (23.2)		
	4	33 (32.4)	24 (31.6)	31 (34.8)	20 (35.7)	< 0.01	0.67
N-stage	0	62 (60.8)	51 (67.1)	40 (44.9)	18 (32.1)		
	1	20 (19.6)	6 (7.9)	14 (15.7)	7 (12.5)		
	2	20 (19.6)	19 (25.0)	35 (39.3)	30 (53.6)		
	3	0 (0)	0 (0)	0 (0)	1 (1.8)	0.13	0.19
Stage	I	10 (9.8)	18 (23.7)	4 (4.5)	4 (7.1)		
	II	23 (22.5)	17 (22.4)	17 (19.1)	6 (10.7)		
	III	25 (24.5)	6 (7.9)	15 (16.9)	7 (12.5)		
	IV	44 (43.1)	35 (46.1)	53 (59.6)	39 (69.6)	0.01	0.4
Vendor	GE	49 (48.0)	0 (0)	70 (78.7)	0 (0)		
	Philips	4 (3.9)	76 (100)	1 (1.1)	55 (98.2)		
	Siemens	48 (47.1)	0 (0)	18 (20.2)	1 (1.8)		
	Toshiba	1 (1.0)	0 (0)	0 (0)	0 (0)	< 0.001	< 0.001
Magnetic field strength	1.0 T	12 (11.8)	0 (0)	1 (1.1)	0 (0)		
	1.5 T	83 (81.4)	58 (76.3)	71 (79.8)	21 (37.5)		
	3.0 T	7 (6.9)	18 (23.7)	17 (19.1)	35 (62.5)	< 0.001	< 0.001
Survival	Deceased	49 (48.0)	24 (31.6)	47 (52.8)	28 (50.0)		
	Alive	53 (52.0)	52 (68.4)	42 (47.2)	28 (50.0)	0.01	0.87
Median time to death	Years (MAD)	1.4 (1.2)	1.3 (1.0)	2.1 (1.9)	2.0 (1.5)	0.03	0.17
Median follow-up time (alive patients)	Years (MAD)	4.5 (2.0)	3.7 (0.9)	5.9 (1.7)	5.0 (0.5)	< 0.001	< 0.001

*Abbreviations*: *MAD*, median absolute deviation; *OPSCC*, oropharyngeal squamous cell carcinoma; *OSCC*, oral cavity squamous cell carcinoma; *T*, Tesla

*p* value* = VUMC OSCC compared to UMCU OSCC, and calculated with the use of Student’s *t* test for continuous variables and *χ*^2^ test for categorical variables

*p* value± = VUMC OPSCC compared to UMCU OPSCC, and calculated with the use of Student’s *t* test for continuous variables and *χ*^2^ test for categorical variables

## Results

### Patient characteristics

Patient cohorts consisted of 102 patients (VUMC OSCC), 76 patients (UMCU OSCC), 89 patients (VUMC OPSCC), and 56 patients (UMCU OPSCC). Patient characteristics for each cohort are presented in Table 2. VUMC OSCC and UMCU OSCC cohorts had similar distributions of age and gender, but VUMC patients presented with higher comorbidity scores (*p* < 0.001), more advanced T-stage (*p* < 0.01), and consequently a poorer overall survival (*p* = 0.01). In contrast, VUMC OPSCC and UMCU OPSCC cohorts only differed significantly from each other in terms of ACE-27 score (*p* = 0.01). Moreover, the scans were obtained using scanners of different vendors and protocols (see also Supplemental Table 1).

### Normalization

**Fig. 3:**
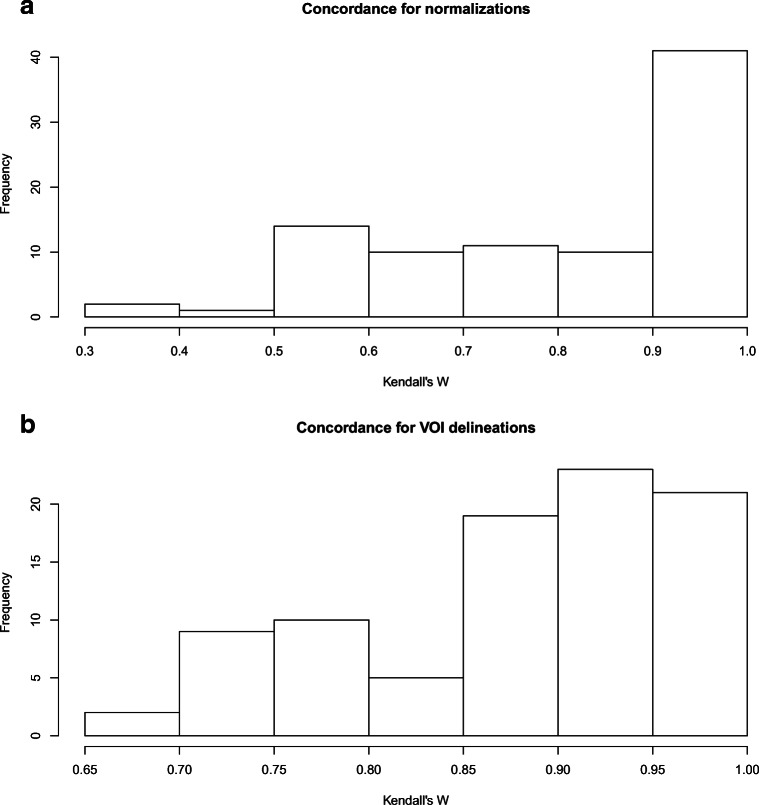
Radiomic features showed high concordance before and after gray level normalization and high interobserver stability. **a** Five methods of gray level normalization were performed before feature extraction and the concordance was calculated of the 89 averaged radiomics features before and after normalization. The figure shows an histogram of the Kendall’s coefficients of concordance (mean = 0.82, sd = 0.19). **b** For 30 VUMC OPSCCs, interobserver stability was assessed by delineation of the tumors by two independent radiologists. The figure shows an histogram of the Kendall’s coefficients of concordance (mean = 0.88, sd = 0.09)

Since different MRI parameters were used on MRI systems supplied by different vendors, we assessed the influence of signal intensities on radiomic analysis [31, 32] using five gray level normalization methods that are described in the Supplemental Methods. A high concordance was found for the 89 radiomics features before and after normalization (mean = 0.82, sd = 0.19). Figure 3 a shows a histogram of the concordances of the core 89 radiomics features. Given the minor influence of gray level normalization on these features, it was decided to proceed with unnormalized data.

### Interobserver stability

Another putative important variable in radiomics feature extraction is definition of the tumor contours by manual delineation, which may introduce variability in the data by inconsistency of segmentation [33]. Therefore, the stability of the radiomics features of a random subgroup of 30 VUMC OPSCCs was assessed when the tumors were delineated by two independent radiologists. A high concordance was found of the 89 radiomics features (mean = 0.88, sd = 0.09) suggesting that delineation by experienced radiologists is consistent or minor changes in delineation do not impact radiomic features. Figure 3b displays the concordances of the 89 core radiomics features with multiple delineations.

### Dimension reduction and factor analysis

Redundancy filtering was applied to the 89 core radiomic features to remove highly correlated features which resulted in 50 features (VUMC OSCC dataset) and 51 features (VUMC OPSCC dataset). A regularized estimator of the correlation matrix between the features was obtained, and factor analysis was performed on this matrix, which showed that both VUMC OSCC features and VUMC OPSCC features were described by 7 latent factors. The factors accounted for 78% (VUMC OSCC) and 77% (VUMC OPSCC) of the variation in the data. The 7 factors can be roughly interpreted as representing (i) 3D geometrics, (ii) meta-gray level co-occurrence, (iii) meta-first order, (iv) gray level mix, (v) meta-gray level run length, (vi) geometrics, and (vii) entropy. The exact content of each factor is shown in Supplemental Table 2 (OSCC) and Supplemental Table 3 (OPSCC). The highest variation in both datasets is explained by factors 1 (3D geometrics) and 2 (meta-gray level co-occurrence).

### OSCC prognostic models

**Fig. 4:**
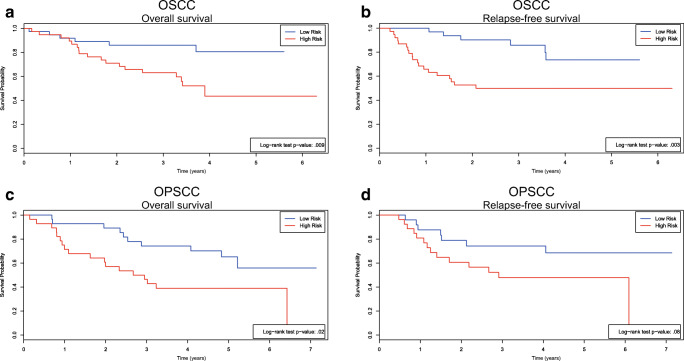
The radiomic signature predicts overall and relapse-free survival in oral cavity squamous cell carcinoma and oropharyngeal squamous cell carcinoma. **a**, **b** Kaplan-Meier analysis of overall survival (**a**) and relapse-free survival (**b**) with risk groups defined by median predicted hazards of the radiomic signature in the UMCU validation cohort of 76 OSCC patients. **c**, **d** Kaplan-Meier analysis of overall survival (**c**) and relapse-free survival (**d**) of different risk groups defined by median predicted hazards of the radiomic signature in the UMCU validation cohort of 56 OPSCC patients. All *p* values are calculated using a log-rank test. Tick marks on curves indicate censoring. Abbreviations: OPSCC, oropharyngeal squamous cell carcinoma; OSCC, oral cavity squamous cell carcinoma

The 7 extracted latent factors were used to train a model to predict OS and RFS of OSCC patients. For OS, an iAUC was found of 0.69 in both the VUMC OSCC cohort and the UMCU OSCC cohort (Table 3). For RFS, iAUCs of 0.63 and 0.70 were found in the VUMC OSCC cohort and the UMCU OSCC cohort, respectively (Table 3). These radiomics models were compared to models using (i) tumor volume, and (ii) clinical variables (N-stage, age at diagnosis and gender). Tumor volume only had a limited prognostic value (iAUC 0.50–0.60). Compared to the radiomics only model, the clinical models performed equally or worse (Table 3). Subsequently, the radiomics and clinical models were combined to assess whether this could further improve the performance. Indeed, the most accurate models were found when radiomics and clinical data were combined (Table 3), and the iAUC improvement was also statistically significant (Supplemental Table 4). Figure 4a and b show Kaplan-Meier curves of the UMCU OSCC cohort with group stratification based on the median predicted risk.

**Table 3:** Performance of radiomic, clinical, and combined models in OSCC and OPSCC cohorts

		Overall survival		Relapse-free survival	
		iAUC (95% CI^a^)	*p*^b^ value	iAUC (95% CI^a^)	*p*^b^ value
OSCC VUMC—training					
Radiomic		0.69 (0.59–0.73)		0.63 (0.50–0.68)	
Clinical^c^		0.69 (0.61–0.75)		0.60 (0.49–0.66)	
Radiomic + clinical^c^		0.75 (0.65–0.77)		0.65 (0.51–0.67)	
OSCC UMCU—validation					
Radiomic		0.69 (0.52–0.75)	0.009	0.70 (0.54–0.75)	0.003
Clinical^c,d^		0.65 (0.51–0.72)	0.02	0.64 (0.51–0.70)	0.08
Radiomic + clinical^c,d^		0.72 (0.55–0.74)	0.01	0.74 (0.58–0.78)	< 0.001
OPSCC VUMC—training					
Radiomic		0.71 (0.62–0.76)		0.70 (0.58–0.77)	
Clinical^c^		0.57 (0.46–0.61)		0.56 (0.42–0.61)	
Radiomic + clinical^c^		0.73 (0.62–0.76)		0.70 (0.56–0.75)	
OPSCC UMCU—validation					
Radiomic		0.71 (0.58–0.77)	0.02	0.74 (0.60–0.83)	0.08
Clinical^c,d^		0.74 (0.64–0.83)	< 0.001	0.71 (0.58–0.82)	0.01
Radiomic + clinical^c,d^		0.81 (0.68–0.91)	< 0.001	0.78 (0.62–0.83)	0.04

*Abbreviations*: *CI*, confidence interval; *iAUC*, integrated area under the curve; *OPSCC*, oropharyngeal squamous cell carcinoma; *OSCC*, oral cavity squamous cell carcinoma

^a^CIs were assessed by bootstrapping

^b^Assessed by log-rank testing in validation cohorts with group stratification based on the median predicted risk

^c^Clinical models consisted of N-stage, age at diagnosis and gender

^d^Recalibration of coefficients of clinical variables was allowed to optimize comparability with radiomic models

For delineation, STIR imaging was also used since the tumors are more clearly discriminated from normal tissue on this sequence. Radiomic features extracted from this sequence may also further improve the prognostic model, and therefore additional prognostic models based on the combination of STIR and T1W MRI radiomic features were trained and validated. In the training cohort, the iAUC did not improve by using the combination of T1W MRI and STIR (Table 4), whereas in the validation cohort the iAUC did improve, but the precision of the estimated iAUC is low given the wide confidence intervals. The difference between the cohorts might also be explained by the shorter follow-up time in the OSCC UMCU cohort or the smaller cohort size (Table 2).

**Table 4:** Performance of radiomic (T1W + STIR), clinical, and combined models in OSCC cohort

		Overall survival		Relapse-free survival	
		iAUC (95% CI^a^)	*p*^b^ value	iAUC (95% CI^a^)	*p*^b^ value
OSCC VUMC—training					
Radiomic		0.67 (0.57–0.71)		0.62 (0.47–0.65)	
Clinical^c^		0.69 (0.61–0.75)		0.60 (0.49–0.66)	
Radiomic + clinical^c^		0.74 (0.64–0.76)		0.65 (0.49–0.66)	
OSCC UMCU—validation					
Radiomic		0.80 (0.68–0.84)	< 0.001	0.72 (0.57–0.77)	0.01
Clinical^c,d^		0.65 (0.51–0.72)	0.02	0.64 (0.51–0.70)	0.08
Radiomic + clinical^c,d^		0.82 (0.67–0.83)	< 0.001	0.76 (0.61–0.80)	0.001

*Abbreviations*: *CI*, confidence interval; *iAUC*, integrated area under the curve; *OPSCC*, oropharyngeal squamous cell carcinoma; *OSCC*, oral cavity squamous cell carcinoma; *STIR*, short TI inversion recovery; T1W, T1-weighted

^a^CIs were assessed by bootstrapping

^b^Assessed by log-rank testing in validation cohorts with group stratification based on the median predicted risk

^c^Clinical models consisted of N-stage, age at diagnosis and gender

^d^Recalibration of coefficients of clinical variables was allowed to optimize comparability with radiomic models

### OPSCC prognostic models

Following the strategy of assessing the relevance of radiomics models in OSCC patients, OPSCC models were trained using radiomics, clinical data, tumor volume, and a combination of both. Note that the study encompassed only HPV-negative cases. Similarly to the OSCC cohorts, radiomics-only models predicted the outcome of OPSCC patients (Table 3). The clinical models, however, were less informative in the VUMC cohort (Table 3). The better performance of the clinical models in the OPSCC UMCU cohort may relate to the shorter follow-up time or the smaller cohort size (Table 2). The combined models showed the highest iAUCs (Table 3), and were significantly better than radiomic and clinical models (Supplemental Table 4). Tumor volume only had a limited prognostic value (iAUC 0.53–0.64). Figure 4c and d show Kaplan-Meier curves of the UMCU OPSCC cohort with group stratification based on the median predicted risk.

### Influence of vendor and magnetic field strength

Radiomic features were extracted from scans with three different magnetic field strengths (Table 2). The VUMC cohorts also consisted of data extracted from scanners of various MR vendors (Table 2). MANOVA analysis implied that there might be an effect of the field strength on factor 3 (meta-first order), factor 4 (gray level mix), and factor 5 (meta-gray level run length) (Supplemental Table 5). Second, MANOVA analysis presented a possible effect of MR vendor on factor 3 (meta-first order), factor 4 (gray level mix), factor 5 (meta-gray level run length), factor 6 (geometrics), and factor 7 (entropy) (Supplemental Table 6). However, the indicated effects were not consistent across datasets, except for factor 3 (meta-first order).

## Discussion

This study was set out to develop prognostic models based on MRI radiomics in oral cavity and oropharyngeal cancer patients. Although MRI is most commonly used in head and neck cancer imaging, clinical routine shows a large variety of MRI vendors and MRI acquisition protocols, which might hamper radiomic analyses. Here we show that despite this potential problem, relevant information can be extracted.

In four patient cohorts, 545 quantitative features were extracted from native T1W MRI, and a four-step method was applied to reduce dimensions while preserving the data’s covariation [30]. This method includes redundancy filtering and factor analysis, and provided models based on 7 latent factors both in OSCC and in OPSCC. These factors roughly describe tumor intensity (i.e., “graylevel-mix” and “meta-firstorder”), shape (i.e., “3D geometrics” and “geometrics”), and texture (i.e., “meta-graylevelco-occurrence,” “meta-graylevelrunlength,” and “entropy”). In validation setting, the prognostic performance of these models was accurate, and the combined models outperformed clinical characteristics alone in predicting both OS and RFS. These results are very promising and indicate that MRI radiomic analysis may have additional value to current prognostic variables.

Furthermore, as with all prognostic models, it is important that it applies in settings outside the reference hospitals involved in the development. Partly, this was overcome by using independent validation cohorts provided by a second institution that uses imaging equipment from different vendors. Moreover, feature stability remained high with and without gray level normalization, and did not depend on interobserver variability. Together this suggests that the external validity of the signature described is expected to be high.

To date, only few prognostic MRI radiomic signatures for HNSCC have been published [20–23]. Most previous studies applied radiomic analyses to CT scans of HNSCC patients [10–13], and comparable performance of the prognostic models was reported. However, in these studies, delineated CT scans from radiotherapy treatment plans were used, which are often not available in surgically treated patients and thereby not available for many HNSCC patients. Nonetheless, MRI radiomics has been applied to nasopharyngeal carcinoma [34–39], which is a separate disease entity [40].

Next to radiomic signatures, there is a myriad of other prognostic biomarkers for HNSCC available that, for instance, are based on imaging [41], immunohistochemistry [42], and microarray data [4]. The advantages of our radiomic profile is that it is available before treatment and based on standard diagnostic images, thereby avoiding additional costs and discomfort for the patient. Moreover, radiomic analyses may better capture tumor heterogeneity than biomarkers [43].

Our study has several strengths. First, standard-of-care native T1W MR images were used to extract the radiomic features. This sequence is used in almost all clinical HNSCC protocols and makes the results broadly applicable. Second, multiple adequately sized patient cohorts were imaged on scanners of different vendors to develop and validate the models, which further contributes to the generalizability of the approach. In addition, features were not very sensitive to delineation. Finally, the prognostic signature is interpretable for clinicians: the latent factors represented different tumor characteristics and were subsequently used in Cox regression. Cox regression is familiar to most clinicians as opposed to machine learning algorithms [44], alleviating the “black box” effect of many high-throughput prognostic models.

However, there are also limitations to be identified in this study. Foremost, the MRI scans of the tumors in this study stem from scanners of different vendors and were attained with different acquisition settings, causing data variability. Indeed, our analyses indicate that some factor scores might be influenced by the variety of scanning protocols and used MR equipment. This is especially true for factor 3, which is made up of features describing first-order statistics that would be expected to be influenced by acquisition settings and magnetic field strength. However, the largest variability in the data was explained by factors 1 and 2, which appeared not to be influenced by vendor and field strength variability. Nonetheless, more uniform data will likely improve model performance and validity [8]. Finally, the radiomic signatures were combined with several important clinical variables (e.g., N-stage, age at diagnosis), but combination with other important clinical factors such as smoking (packyears) and alcohol consumption (unityears) might improve prediction accuracy further [45]. Of note, the retrospective nature of this study precluded the use of the 8th edition of the UICC TNM Classification because important information was not available (i.e., clinical depth of invasion and clinical extranodal extension). However, it has been shown that the 8th edition outperforms the previous edition [46], and including the new system in future studies may improve prediction of the clinical and combined clinical-radiomic models.

In conclusion, we developed and validated a prognostic signature based on radiomic features extracted from standard-of-care MRI. This finding suggests that important prognostic information is present in MRI databases of HNSCC patients across the world. It also implicates that MRI acquisition protocols should be further standardized to optimize exchangeability of data and models. Future research could focus on analysis of feature stability by scanning patients on scanners of different vendors, and on the same scanner at multiple time points (test-retest analysis). Moreover, we already show that combining multiple sequences may improve the prognostic performance of the model, while future studies should incorporate functional MRI sequences and multiple imaging modalities (i.e., CT and PET) to capture more aspects of tumor biology.

## Electronic supplementary material

ESM 1 (DOCX 30 kb)

ESM 2 (XLSX 35 kb)

ESM 3 (XLSX 12 kb)

ESM 4 (XLSX 10 kb)

ESM 5 (XLSX 10 kb)

ESM 6 (XLSX 11 kb)

ESM 7 (XLSX 11 kb)

## Abbreviations

ACE-27: Adult Comorbidity Evaluation 27
HNSCC: Head and neck squamous cell carcinoma
iAUC: Integrated area under the curve
MANOVA: Multivariate analysis of variance
OPSCC: Oropharyngeal squamous cell carcinoma
OS: Overall survival
OSCC: Oral squamous cell carcinoma
RFS: Relapse-free survival
STIR: Short TI inversion recovery
