# Supplementary material for: Outcome prediction of head and neck squamous cell carcinoma by MRI radiomic signatures
Source: Eur Radiol. 2020 Jun 4;30(11):6311–21. doi: 10.1007/s00330-020-06962-y (PMC7554007; doi:10.1007/s00330-020-06962-y)
Supplement: Supplementary file 1 — (DOCX 30 kb) [file 330_2020_6962_MOESM1_ESM.docx]

**Supplemental methods**

*Feature extraction*

In total, 545 radiomic features were extracted from the tumor VOIs projected onto the T1W images using ACCURATE [1; 2], which is available at <https://petralymphoma.org/accurate-tool/>. This version was modified to allow DICOM input of MRI. No voxel interpolation was applied before feature extraction. The features were either based on first order statistics (n=35), intensity-volume histograms (n=1), morphology (n=11), fractals (n=4), spatial autocorrelation (n=2) or texture (n=492). Texture features were derived from gray-level co-occurrence matrices and from gray-level run-length matrices. Each texture feature was calculated in four ways. For the first approach, matrices were created per direction and per x-y plane (2D) for all planes and all four possible directions, and subsequently combined into a single matrix that was used to calculate the feature from (2D combined). For the second approach, the feature was calculated from each matrix, created per direction and per x-y plane (2D) for all planes and all four possible directions, and then averaged (2D averaged). For the third approach, matrices were created per direction for the entire VOI (3D) for all thirteen possible directions, and subsequently combined into a single matrix that was used to calculate the feature from (3D combined). For the fourth approach, the feature was calculated from each matrix, created per direction for all thirteen possible directions, and then averaged (3D averaged). In addition, a discretization of 32, 64 or 128 gray level bins was applied (Table 1). All features were implemented according to the recommendations set by the image biomarker standardization initiative [3].

*Normalization*

Because various MRI parameters were used including intensity features, a gray level normalization was applied to the MRI scans in advance of feature extraction. Four schemes for normalization were considered:

- N-I: no normalization was applied.
- N-II: a multiplicative transformation was used to fix the range of gray levels for all the images [4]. Each gray level was multiplied by the ratio MEDIAN/(median of the reference volume of interest (VOI)) where MEDIAN was a constant, i.e. the median value for all analyzed reference VOIs.
- N-III: the gray levels within the tumor VOI that were located outside the range of three standard deviations of the average voxel intensity were not considered in further analysis [4].
- N-IV: the median value of the reference VOI was subtracted from gray levels within the tumor VOI and subsequently divided by the median absolute deviation (MAD) within the reference VOI according to the following formula:

$$I_{N}=\frac{I-median(I_{ref})}{{MAD}_{ref}}$$

Where *I_N_* is the normalized gray level within the tumor VOI, *I* is the gray level within the tumor VOI in advance of normalization, *I_ref_* is the gray level within the reference VOI and *MAD_ref_* is the MAD within the reference VOI.

For N-II and N-IV, two reference structures were used for normalization, i.e. the obliquus capitis inferior muscle (N-IIa and N-IVa) and the myelum (N-IIb and N-IVb).

*Feature processing*

As described before, texture features were calculated using different settings for coding of gray level intensity, different numbers of considered directions and different methods of including the obtained values in the considered directions. The average value of similar features with different calculation settings was determined reducing the initial 545 raw radiomic features to 89 core features (see also Table 1). These core features were subsequently used in downstream analysis (see *Factor analysis*).

*Factor analysis*

After obtaining the core 89 features, redundancy filtering was performed by removing the minimal number of features under marginal correlation threshold τ, which was set τ=0.95. Next, features were scaled (centered around 0 and variance 1) to avoid a situation where the features with the largest scale dominate the analysis. A regularized estimator of the correlation matrix between the scaled features was obtained, and a maximum likelihood factor analysis was performed on the matrix. The number of latent features was determined using the Guttman-Kaiser rule [5] on the regularized correlation matrix. Factor scores were obtained by regressing the latent features on the observed data by way of the obtained factor solution. All steps were carried out using the R package "FMradio: Factor modeling for radiomic data”, version 1.1.1 (<https://CRAN.R-project.org/package=FMradio>)[6].

*Prognostic model training*

The resulting factor scores were used as predictors for a Cox model built on the training sets: (1) VUMC OSCC, and (2) VUMC OPSCC. Subsequently, the parameter matrices of the factor analysis performed on the training sets were used to construct factor scores for the samples in the validation sets: (1) UMCU OSCC, and (2) UMCU OPSCC, and these factor scores were used as predictors in a Cox model for the validation sets. Models were built to predict overall and disease-free survival. Performance of all models was assessed in terms of the (integrated) area-under-the-curve (iAUC) with a confidence interval that was assessed by bootstrapping (1000 times). Moreover, the patients of the validation sets were divided into low-risk and high-risk groups based on the median of the model scores and a log-rank test was performed. The radiomics only models were compared to a clinical-variables only model and a model holding both the radiomic features and clinical-variables. Clinical models were based on those features that were uniformly available and for which, *a priori,* some predictive power is expected: i.e. N-stage, age at diagnosis, and gender. T-stage and ACE-27 score held no predictive power in this dataset. Additionally, the radiomics only models were compared to tumor volume alone as proposed by Welch et al [7]. The iAUC of the combined model (radiomics + clinical) was compared to the iAUC of the individual prognostic models (radiomics only + clinical only) with the Wilcoxon rank sum test for dependent samples [8], and a multiplicity correction was performed using the Holm method. P-values of less than 0.05 were considered statistically significant.

**References**

1 van Velden FH, Kramer GM, Frings V et al (2016) Repeatability of Radiomic Features in Non-Small-Cell Lung Cancer [(18)F]FDG-PET/CT Studies: Impact of Reconstruction and Delineation. Mol Imaging Biol 18:788-795

2 Boellaard R (2018) Quantitative oncology molecularanalysis suite: ACCURATE. Journal of Nuclear Medicine 59

3 Zwanenburg A, Leger S, Vallières M, Löck S (2016) Image biomarker standardisation initiative. arXiv:1612.07003 [cs.CV]

4 Collewet G, Strzelecki M, Mariette F (2004) Influence of MRI acquisition protocols and image intensity normalization methods on texture classification. Magn Reson Imaging 22:81-91

5 Kaiser HF (1970) A second generation little jiffy. Psychometrika 35:401-415

6 Peeters CFW, Übelhör C, Mes SW et al (2019) Stable prediction with radiomics data. arXiv:1903.11696 [stat.ML]

7 Welch ML, McIntosh C, Haibe-Kains B et al (2019) Vulnerabilities of radiomic signature development: The need for safeguards. Radiotherapy and Oncology 130:2-9

8 Haibe-Kains B, Desmedt C, Sotiriou C, Bontempi G (2008) A comparative study of survival models for breast cancer prognostication based on microarray data: does a single gene beat them all? Bioinformatics 24:2200-2208
